# Validation of the Relationship Between Iris Color and Uveal Melanoma Using Artificial Intelligence With Multiple Paths in a Large Chinese Population

**DOI:** 10.3389/fcell.2021.713209

**Published:** 2021-08-19

**Authors:** Haihan Zhang, Yueming Liu, Kai Zhang, Shiqi Hui, Yu Feng, Jingting Luo, Yang Li, Wenbin Wei

**Affiliations:** ^1^Beijing Tongren Eye Center, Beijing Key Laboratory of Intraocular Tumor Diagnosis and Treatment, Beijing Ophthalmology and Visual Sciences Key Lab, Medical Artificial Intelligence Research and Verification Key Laboratory of the Ministry of Industry and Information Technology, Beijing Tongren Hospital, Capital Medical University, Beijing, China; ^2^SenseTime Group Ltd., Shanghai, China

**Keywords:** uveal melanoma, iris color, artificial intelligence, machine learning, Chinese population

## Abstract

Previous studies have shown that light iris color is a predisposing factor for the development of uveal melanoma (UM) in a population of Caucasian ancestry. However, in all these studies, a remarkably low percentage of patients have brown eyes, so we applied deep learning methods to investigate the correlation between iris color and the prevalence of UM in the Chinese population. All anterior segment photos were automatically segmented with U-NET, and only the iris regions were retained. Then the iris was analyzed with machine learning methods (random forests and convolutional neural networks) to obtain the corresponding iris color spectra (classification probability). We obtained satisfactory segmentation results with high consistency with those from experts. The iris color spectrum is consistent with the raters’ view, but there is no significant correlation with UM incidence.

## Introduction

Uveal melanoma (UM) is the most common primary intraocular malignancy in adults. In a study by Shields et al. (2009) of 8,033 UM patients, tumors were located in the iris in 285 cases (4%), the ciliary body in 492 cases (6%), and the choroid in 7,256 cases (90%). About half of patients eventually developed blood metastases, often on the liver. Most tumors can be treated by irradiation (e.g., radioactive plaque, proton beam), and larger tumors may require eye excision (Dogrusöz et al., 2017). The main goal of treatment is to locally control tumor growth and prevent tumor metastasis and spreading. There is currently no effective treatment for metastasis. Consequently, the vast majority of patients die in a short period (6–8 months) (Singh et al., 2005; Cerbone et al., 2014). UM is primarily diagnosed by clinical examination, including indirect ophthalmoscopy and ancillary examinations such as fluorescent angiography and ophthalmic ultrasound. However, many patients come to the doctor late because they have no symptoms. When patients have symptoms, they often suffer blurred vision, light patches (seeing flashes of light), visual field defects, etc., (Damato and Damato, 2012).

The annual incidence of UM is 6 per 1 million people (Boyle et al., 1983). Among a host of factors associated with an increased incidence of UM, ethnicity is the strongest risk factor for UM. UM is approximately 20–30 times more common in whites than in blacks and Asians. Among whites, light skin color and light iris color are established risk factors (Gallagher et al., 1985; Holly et al., 1990; Vajdic et al., 2002; Shah et al., 2005). In a meta-analysis of the association between host susceptibility factors and UM presented by Weis et al. (2006), the following statistically significant factors were revealed: light eye color (RR, 1.75), light skin color (RR, 1.80), and inability to tan (RR, 1.64). The increased incidence of UM in eyes with light iris (blue or gray) may be associated with a decrease in uveal melanin. Iris pigmentation has many physiological functions, including protection of the underlying tissues from ultraviolet radiation, and a protective role in various diseases (e.g., age-related macular degeneration, age-related cataract) (Cumming et al., 2000; Tomany et al., 2003). Lack of pigmentation leads to more light penetration into the uvea and less protection from ultraviolet radiation (UV), which increases the risk of UM (Egan et al., 1988; Singh and Topham, 2003). It has also been suggested (Houtzagers et al., 2020) that most UV rays are absorbed by the cornea, lens, and vitreous, while other wavelengths, such as visible light, penetrate the back of the eye and contribute to the production of toxic reactive oxygen species (ROS), thereby increasing the chance of malignant transformation of uveal melanocytes.

Although several studies from Canada, the United States, Germany, France, the Netherlands, and Australia have shown that UM is more prevalent in people with lighter iris color (Gallagher et al., 1985; Holly et al., 1990; Seddon et al., 1990; Pane and Hirst, 2000; Guénel et al., 2001; Stang et al., 2003; Schmidt-Pokrzywniak et al., 2009; Houtzagers et al., 2020), the proportion of patients with brown eyes in these studies is very low. Therefore, the relationship between iris color and the incidence of UM in the Asian population requires a closer examination. For the first time, we applied deep learning methods to retrospectively evaluate the iris color of the eyes with UM using the photos from Chinese patients, to investigate the correlation between iris color and the prevalence of UM in the East Asian population.

## Materials and Methods

### Study Population

**FIGURE 1:**
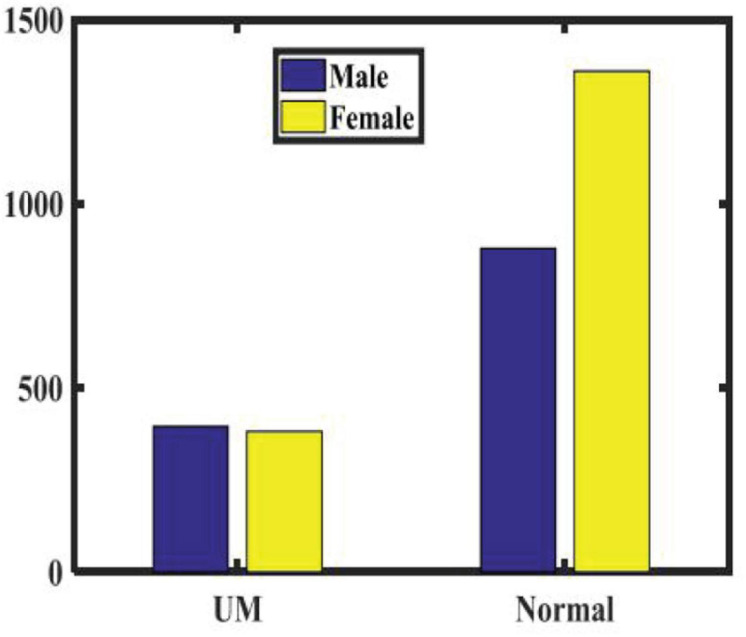
Sex ratio of patients in the two groups.

**FIGURE 2:**
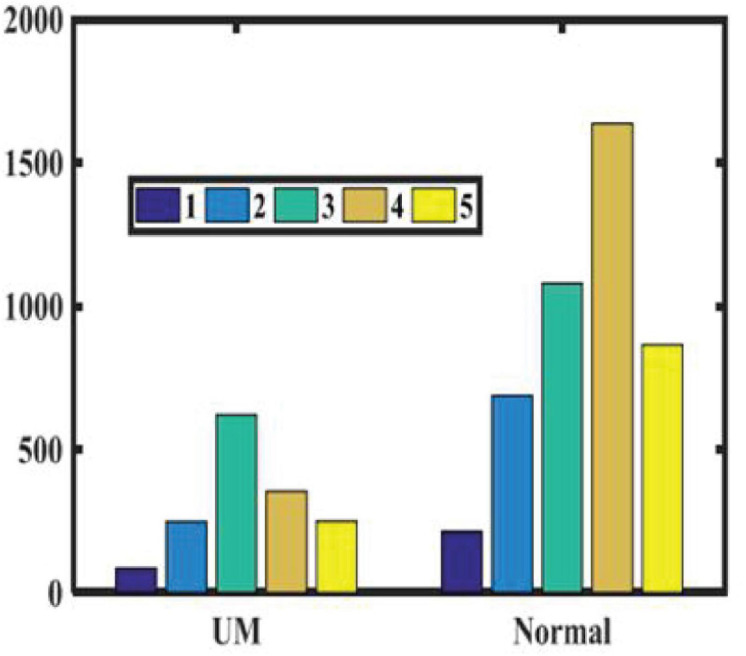
Iris color grading of patients in two groups.

**FIGURE 3:**
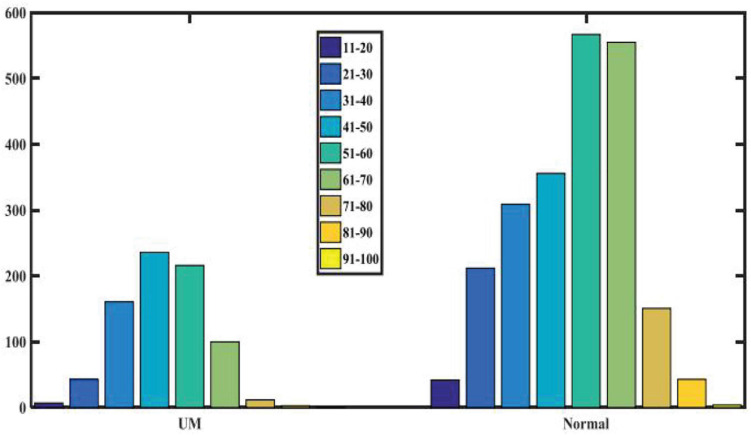
Age distribution of patients in the two groups.

**FIGURE 4:**
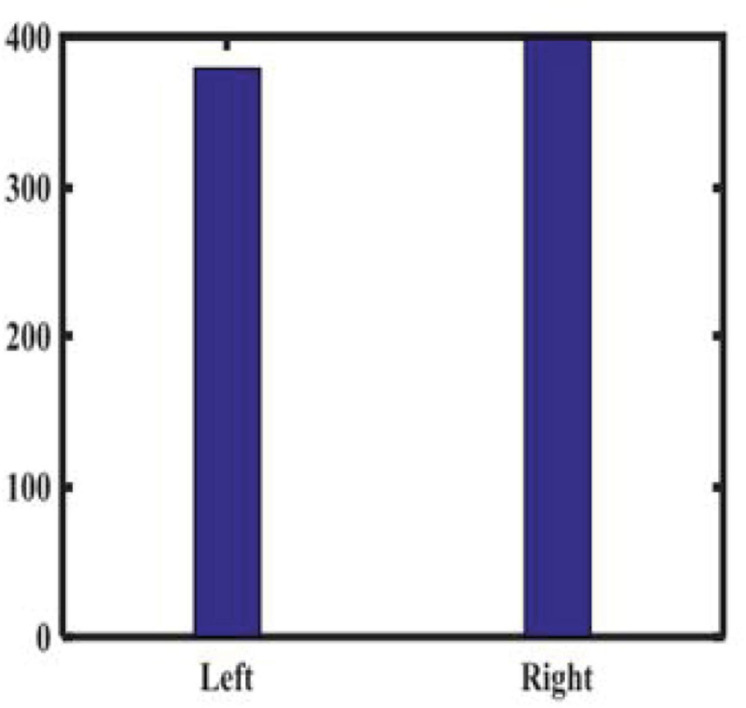
The laterality of the affected eye of uveal melanoma (UM) patients.

We randomly recruited patients with benign eye diseases admitted to Beijing Tongren Hospital between 2015 and 2020 as a control group. We excluded eyes with previous iris laser treatment, or under IOP-lowering medication, as these conditions may have changed the iris color or morphology in some eyes. We also excluded some eyes with iris depigmentation or corneal leucoma, which may affect our judgment of iris color. Our study included 778 UM patients and 2,239 nontumor patients, all of which have clearly recognizable anterior segment photos of both eyes. None of the patients received any treatment, including tumor resection, radiotherapy, and chemotherapy, before we took images of their anterior segments. According to patients’ medical records and clinical photos, all patients’ iris colors were divided into five groups. Table 1 shows the baseline characteristics of the study population. As shown in Figure 1, there is no significant difference in the ratio of males to females between the two groups. As shown in Figure 2, in both populations, eyes with an iris color rating of 3 or 4 were the most common. The mean age of UM patients was 48 years, and that of nontumor patients was 52 years. Figure 3 shows the age distribution of the two groups of patients. Figure 4 shows that 379 of the UM patients had tumors in the left eye and 399 in the right eye, with no significant difference in the affected eye. The detailed information of the included patients is shown in Supplementary Tables 1, 2.

**TABLE 1:** Baseline characteristics of the study population.

	**UM (*n* = 778)**		**Normal (2,239)**	
	**n**	**%**	***N***	**%**
**Gender**				
Male	396	0.51	879	0.39
Female	382	0.49	1360	0.61
**Age**				
Mean	48.0		52.0	
**Iris Color**	**1,556**		**4,478**	
➀	85	0.055	212	0.047
➁	248	0.159	687	0.153
➂	620	0.398	1079	0.241
➃	354	0.228	1636	0.365
➄	249	0.160	864	0.193

### Iris Color Grading

A retrospective assessment of iris color was performed by using anterior segment photos of UM patients and nontumor patients at their first visit to Beijing Tongren Hospital from 2015 to 2020. Color images of the iris of both eyes were taken using a slit lamp (DC3, Topcon Corporation, Tokyo, Japan) with a × 16 magnification, bandwidth (>20 mm), height (14 mm), brightness at 30% of the brightest, and angle of 45°. Shot in a darkened room (20 lux), photos are saved in JPEG format (RGB 3120 × 4160) (ACDSee Photo Manager Version 11.0 Software View)^1^.

The iris color-grading scheme in our study is the same as described in previous studies of Asians (Sidhartha et al., 2014a,b; Pan et al., 2018). The participants’ demographic information and clinical diagnosis were masked, and the color of all iris photos was rated independently by two raters. We select a set of reference photos that best represent the changes observed in the study population. The iris is rated on a scale of 1–5 based on the overall color of the iris: 1 for the lightest color and 5 for the darkest. If a photograph is considered to be between two consecutive levels, the higher level is assigned. If the two raters’ observations do not agree, a third person makes the judgment.

Our raters all have some medical background and general knowledge of ophthalmology.

### Annotation Site Segmentation From Slit-Lamp Images

**FIGURE 5:**
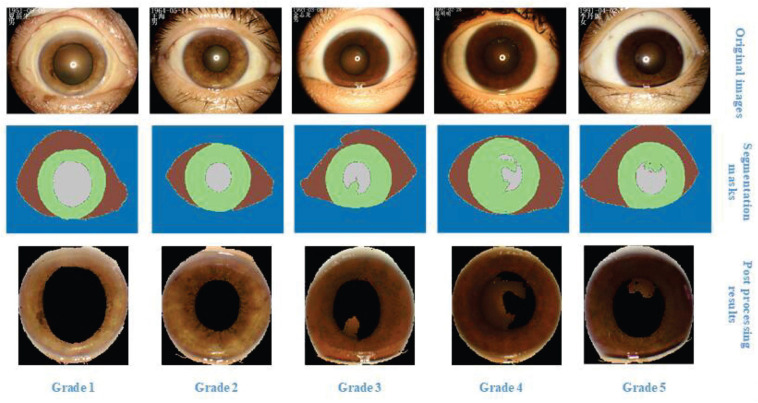
The most representative photos in the current study population were selected as a reference for scoring and the corresponding segmentation results.

We applied U-NET (Ronneberger et al., 2015) to automatically extract iris zones and obtained the post-processed images which only contain iris sites. Then, the segmented mask results were used to detect connected zones and the largest zone was retained and the others were discarded, as demonstrated in Figure 5.

**FIGURE 6:**
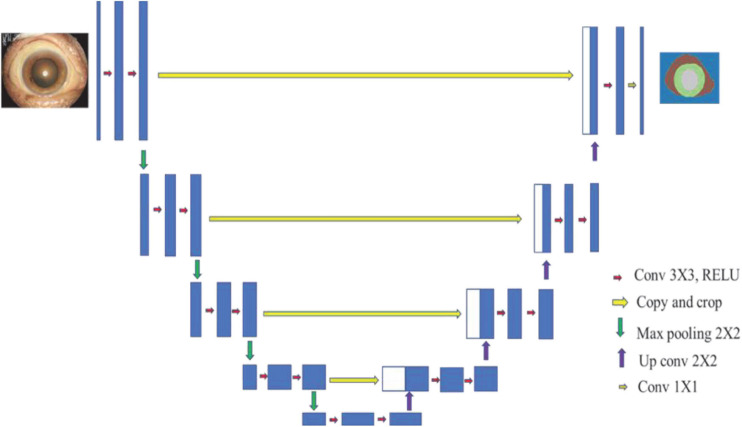
The architecture of U-Net.

The architecture of U-NET is shown in Figure 6. The input and output are the slit-lamp image and mask image, respectively. U-NET can classify all pixels in images to corresponding classes. In the current research, we need to distinguish the pupil, iris, sclera, and eyelid. Then the iris was retained and the pixels in other parts were set as 0. The largest connected region in the slit-lamp image was extracted, and other small components in the iris part are discarded.

### Iris Color Spectrum Extraction and Differentiation of UM Patients’ Images

We used random forest (RF; Zhang et al., 2019; Lin et al., 2020) and convolutional neural network (CNN; Yang et al., 2019; Li et al., 2020; Zhang K. et al., 2020; Zhang Y. et al., 2020) to extract the iris color spectrum, which represents the iris color grading scores of five categories. Then the iris color spectrum was used as the descriptor to differentiate the nontumor patients from UM patients. RF and CNN received the color features and the segmented iris images, respectively. Color features (Shih and Liu, 2016; Wang et al., 2017; Zhang et al., 2018) were computed with RGB (red, green, blue), HSV (hue, saturation, value) (Hamuda et al., 2017), and YCbCr (Noda and Niimi, 2007) color spaces. A total of 27 (three types of color space × three channels × three orders of momentum) features were summarized as the descriptor of the color of iris.

**FIGURE 7:**
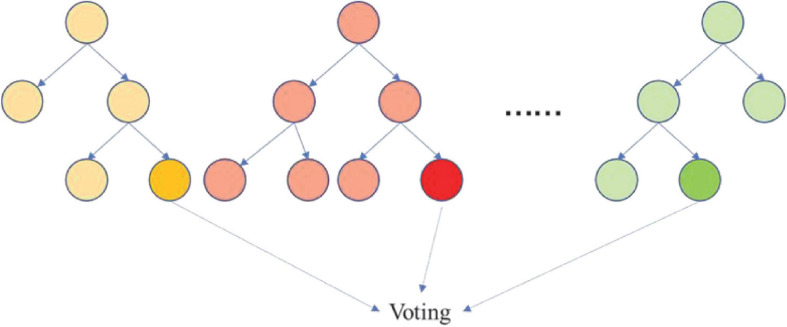
Random forest. The iris color spectrum is defined as the vector in which the five probabilities ([*p*_1_,*p*_2_,*p*_3_,*p*_4_,*p*_5_]) correspond to the five grades of the color of the iris.

The random forest, a common machine learning algorithm, is shown in Figure 7. It consists of many decision trees, and each tree can iteratively split a dataset into subsets as a certain discipline (e.g., Gini index) to complete the classification or regression task. Finally, all trees vote for a specific sample to determine the predictive result.

### Direct Identification of UM With Iris Images

The iris images were directly fed into CNN to test whether it can be used to identify the UM patients from normal persons. The subjects in the training, validation, and testing datasets are different to guarantee the fair evaluation of the relationship between the iris color and UM.

### Statistical Analysis

All statistical analyses were performed using Python 3.7.3 (Wilmington, DE, United States) and MATLAB R2016a.^2^ We used the accuracy, sensitivity, specificity, receiver-operating characteristic curve, and precision recall curve to assess the performance of the machine learning models. The area under the curve (AUC) was calculated.

## Results

**FIGURE 8:**
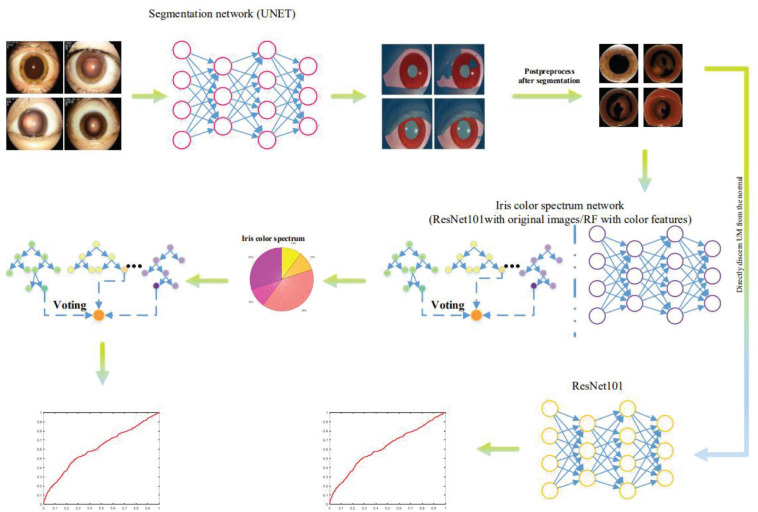
U-NET was used to extract iris regions from slit-lamp images, and then random forest (RF) and convolutional neural network (CNN) were used to extract iris color chromatography as descriptors to distinguish UM patients from nontumor patients. Meanwhile, the extracted iris images were directly input into the CNN network to identify UM patients and nontumor groups.

The loss curve of the UM patient group and the segmentation results are shown in Figure 8. The performance is satisfactory. The iris color spectrum was consistent with the raters’ view. Although the top 1 accuracy is not high, the overall trend is consistent. Because of the physiological limitation and subjectivity of human beings, this result is acceptable and it also verifies that the labels of all samples are objective.

The differentiation results are shown in Figure 8, which is not satisfactory and cannot be discerned by the iris color spectrum. The ROC and PR curves (Wang et al., 2017; Zhang et al., 2018) show that the iris color spectrum almost has no relationship with UM incidence. We also applied the segmented iris map to directly discern whether this patient suffers from UM or not. The performance is also not satisfactory.

**FIGURE 9:**
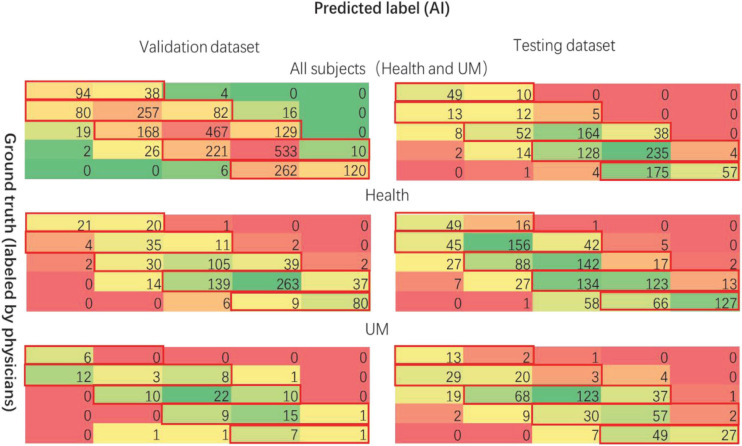
The performance for evaluating the color of iris (RF). The left-hand side is listed as the validation dataset, and the right-hand side is listed as the testing dataset. The first line shows all subjects, the second one shows the nontumor control datasets, the third one shows the tumor patient datasets.

**FIGURE 10:**
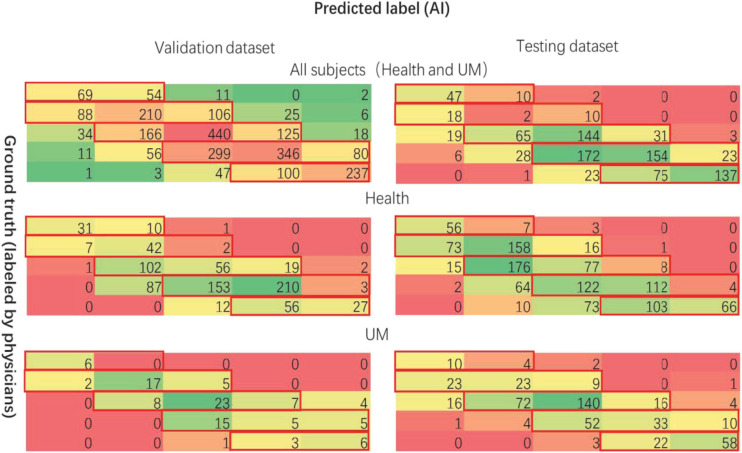
The performance for evaluating the color of the iris (CNN).

**FIGURE 11:**
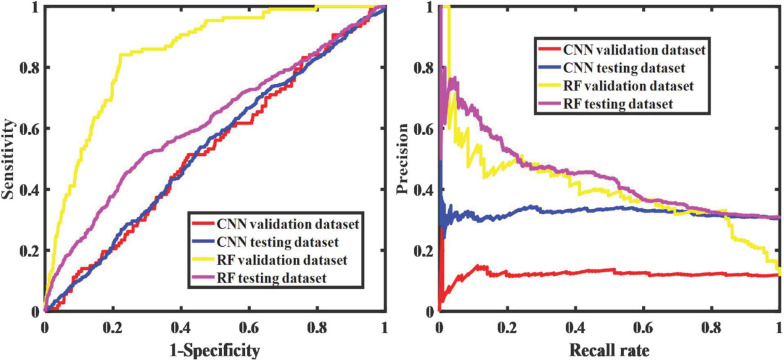
The relationship between the color spectra of the iris and UM.

Figures 9, 10 show the classification performance for evaluating the iris color, including RF and CNN, which distinguishes UM with the color spectrum of the iris and iris image. The results of the RF and CNN classification for iris color are in good agreement with our raters. A small number of different categories of classification results, which are also mainly in the adjacent categories of the raters’ markup results, are shown in the red boxes in Figures 9, 10. Our results show no significant correlation between UM incidence and iris color in our population-based study, as demonstrated in Figure 11. The ROS curves signified that machine learning cannot discern UM with the color features of the iris. Furthermore, we try to directly differentiate UM from the normal with the iris image using CNN, the relationship was also weak.

## Discussion

Uveal melanoma is an aggressive malignancy that originates from melanocytes in the eye and remains to have a poor prognosis with a 5-year overall survival (OS) rate of <50%. The prevalence in Asian populations is about (0.1–0.6)/1,000,000 (Hu et al., 2005; Stang et al., 2005; Park et al., 2015; Tomizuka et al., 2017), which is much lower than that in the non-Hispanic white population (Hu et al., 2005), the highest incidence in Northern Europe (Denmark and Norway) (Schmidt-Pokrzywniak et al., 2009). Regional differences may be attributed to ethnicity (lower risks among Asians and populations with higher levels of melanin production in the iris) or to environmental factors, including UV (Vajdic et al., 2002). In whites, a light iris color is an identified risk factor for UM. Our study is the first to verify the relationship between iris color and UM in East Asian ethnicity using deep learning methods. The results showed that there was no significant correlation between the incidence of UM and iris color.

We compared our findings with other studies that have published cohorts of UM patients with known iris colors (Gallagher et al., 1985; Holly et al., 1990; Seddon et al., 1990; Pane and Hirst, 2000; Guénel et al., 2001; Stang et al., 2003; Schmidt-Pokrzywniak et al., 2009; Houtzagers et al., 2020), the size of our control group (*n* = 2,239) was much larger than in any of the other studies, and the number of UM patients in our study was also relatively large. Compared to the methods used in other studies, deep learning is a more novel and efficient method. In recent years, due to the unique advantages of artificial intelligence in intelligent identification, data mining, information classification, and other aspects, it has brought new scientific research technology to the medical industry, has accelerated the mining of medical information, and has been gradually widely used in the medical field. The accuracy of deep learning for image recognition depends on the number of cases, which often requires a long process of training and learning and optimization. The more image data available for learning, the more accurate the classification results will be. In our study, even though we eliminated many factors that may affect the judgment of iris color, we still included a considerable number of patients and control groups, which is conducive to improving the accuracy of machine learning algorithms.

At the same time, its limitations should be taken into account. First of all, the study population included only that of Chinese so that future studies may address patients of different races. Second, the method of iris color grading was susceptible to subjective factors; therefore, our three raters made their own judgments independently and did not know the classification results of others. Third, it is well known that the observation of color is heavily dependent on light sources; the color rendering index (Ra = color seen under a certain light source/color that can be seen under natural light irradiation) and color temperature are best in natural light, but it is difficult to capture all images in the same natural light. Therefore, we shot in a dark room and used the illumination head LI 900 combined with slit-lamp types BQ 900 BM 900 and BP 900. LI 900 is equipped with two individually adjustable LEDs. The first LED is used for slit illumination and the second for the background illumination. The diffuse light illumination is evenly balanced; the background light and the diffuse light illumination of the slit light gave a free shadow illumination, natural color, and two kinds of reflected light. Besides, since the color temperature and color rendering index of the illumination light sources of the two slit lamps we used were slightly different, and the years of image acquisition span a wide range (5 years), it might have affected the manual judgment of iris color to a certain extent. To solve this problem, we tried to balance the patients included and randomly selected nontumor patients who came to the hospital at the same time as the UM patients.

All in all, the reason why our results differ from previous studies is considered to be mainly the variability of ethnicity. Our study complements the relationship between UM and iris color, laying a foundation for further studies of host susceptibility factors and their molecular mechanisms. We intend to further explore the molecular verification of our research results, and the possible mechanism should be discussed by comparing with the Caucasian ancestry.

## Data Availability Statement

The raw data supporting the conclusions of this article will be made available by the authors, without undue reservation.

## Ethics Statement

Written informed consent was obtained from the individual(s), and minor(s)’ legal guardian/next of kin, for the publication of any potentially identifiable images or data included in this article.

## Author Contributions

HZ, YuL, KZ, YaL, and WW: design of the study. HZ and KZ: development of the algorithm. YuL, YaL, and WW: gathering of the data. HZ, YuL, KZ, SH, YF, and JL: performing of the data analysis. HZ, YuL, and KZ: drafting of the first version of the manuscript. All authors revision and approval of the manuscript.

## Conflict of Interest

KZ was employed by company SenseTime Group Ltd. The remaining authors declare that the research was conducted in the absence of any commercial or financial relationships that could be construed as a potential conflict of interest.

## Publisher’s Note

All claims expressed in this article are solely those of the authors and do not necessarily represent those of their affiliated organizations, or those of the publisher, the editors and the reviewers. Any product that may be evaluated in this article, or claim that may be made by its manufacturer, is not guaranteed or endorsed by the publisher.

## Footnotes

1
https://www.acdsee.com/en/index/

2
https://www.mathworks.com/

## References

[B1] Boyle P. Day N. E. Magnus K. (1983). Mathematical modelling of malignant melanoma trends in Norway, 1953–1978. *Am. J. Epidemiol.* 118 887–896. 10.1093/oxfordjournals.aje.a113706 6650489

[B2] Cerbone L. Van Ginderdeuren R. Van den Oord J. Fieuws S. Spileers W. Van Eenoo L. (2014). Clinical presentation, pathological features and natural course of metastatic UM, an orphan and commonly fatal disease. *Oncology* 86 185–189. 10.1159/000358729 24776955

[B3] Cumming R. G. Mitchell P. Lim R. (2000). Iris color and cataract: the blue mountains eye study. *Am. J. Ophthalmol.* 130 237–238. 10.1016/s0002-9394(00)00479-7 11004303

[B4] Damato E. M. Damato B. E. (2012). Detection and time to treatment of UM in the United Kingdom: an evaluation of 2,384 patients. *Ophthalmology* 119 1582–1589. 10.1016/j.ophtha.2012.01.048 22503229

[B5] Dogrusöz M. Jager M. J. Damato B. (2017). UM treatment and prognostication. *Asia Pac. J. Ophthalmol.* 6 186–196. 10.22608/APO.201734 28399342

[B6] Egan K. M. Seddon J. M. Glynn R. J. Gragoudas E. S. Albert D. M. (1988). Epidemiologic aspects of UM. *Surv. Ophthalmol.* 32 239–251. 3279559 10.1016/0039-6257(88)90173-7

[B7] Gallagher R. P. Elwood J. M. Rootman J. Spinelli J. J. Hill G. B. Threlfall W. J. (1985). Risk factors for ocular melanoma: Western Canada melanoma study. *J. Natl. Cancer Inst.* 74 775–778. 3857374

[B8] Guénel P. Laforest L. Cyr D. Févotte J. Sabroe S. Dufour C. (2001). Occupational risk factors, ultraviolet radiation, and ocular melanoma: a case-control study in France. *Cancer Causes Control.* 12 451–459. 11545460 10.1023/a:1011271420974

[B9] Hamuda E. Mc Ginley B. Glavin M. Jones E. (2017). Automatic crop detection under field conditions using the HSV colour space and morphological operations. *Comput. Electron. Agric.* 133 97–107.

[B10] Holly E. A. Aston D. A. Char D. H. Kristiansen J. J. Ahn D. K. (1990). UM in relation to ultraviolet light exposure and host factors. *Cancer Res.* 50 5773–5777. 2393851

[B11] Houtzagers L. E. Wierenga A. P. A. Ruys A. A. M. Luyten G. P. M. Jager M. J. (2020). Iris colour and the risk of developing UM. *Int. J. Mol. Sci.* 21:7172. 10.3390/ijms21197172 32998469 PMC7583924

[B12] Hu D. N. Yu G. P. McCormick S. A. Schneider S. Finger P. T. (2005). Population-based incidence of uveal melanoma in various races and ethnic groups. *Am. J. Ophthalmol.* 140 612–617. 16226513 10.1016/j.ajo.2005.05.034

[B13] Li W. Yang Y. Zhang K. Long E. He L. Zhang L. (2020). Dense anatomical annotation of slit-lamp images improves the performance of deep learning for the diagnosis of ophthalmic disorders. *Nat. Biomed. Eng.* 4 767–777. 10.1038/s41551-020-0577-y 32572198

[B14] Lin D. Chen J. Lin Z. Li X. Zhang K. Wu X. (2020). A practical model for the identification of congenital cataracts using machine learning. *EBioMedicine* 51:102621. 10.1016/j.ebiom.2019.102621 31901869 PMC6948173

[B15] Noda H. Niimi M. (2007). Colorization in YCbCr color space and its application to JPEG images. *Pattern Recognit.* 40 3714–3720.

[B16] Pan C.-W. Qiu Q.-X. Qian D.-J. Hu D.-N. Li J. Saw S.-M. (2018). Iris colour in relation to myopia among Chinese school-aged children. *Ophthalmic Physiol. Opt.* 38 48–55. 29265474 10.1111/opo.12427

[B17] Pane A. R. Hirst L. W. (2000). Ultraviolet light exposure as a risk factor for ocular melanoma in Queensland, Australia. *Ophthalmic Epidemiol.* 7 159–167. 11035552

[B18] Park S. J. Oh C. M. Kim B. W. Woo S. J. Cho H. Park K. H. (2015). Nationwide incidence of ocular melanoma in South Korea by using the national cancer registry database (1999–2011). *Invest. Ophthalmol. Vis. Sci.* 56 4719–4724. 26207308 10.1167/iovs.15-16532

[B19] Ronneberger O. Fischer P. Brox T. (2015). “U-net: convolutional networks for biomedical image segmentation,” in *Medical Image Computing and Computer-Assisted Intervention–MICCAI 2015. MICCAI 2015. Lecture Notes in Computer Science*, Vol. 9351 eds Navab N. Hornegger J. Wells W. Frangi A. (Cham: Springer). 10.1007/978-3-319-24574-4_28

[B20] Schmidt-Pokrzywniak A. Jöckel K. H. Bornfeld N. Sauerwein W. Stang A. (2009). Positive interaction between light iris color and ultraviolet radiation in relation to the risk of UM: a case-control study. *Ophthalmology* 116 340–348. 19091418 10.1016/j.ophtha.2008.09.040

[B21] Seddon J. M. Gragoudas E. S. Glynn R. J. Egan K. M. Albert D. M. Blitzer P. H. (1990). Host factors, UV radiation, and risk of UM. A case-control study. *Arch. Ophthalmol.* 108 1274–1280. 2400347 10.1001/archopht.1990.01070110090031

[B22] Shah C. P. Weis E. Lajous M. Shields J. A. Shields C. L. (2005). Intermittent and chronic ultraviolet light exposure and UM: a meta-analysis. *Ophthalmology* 112 1599–1607. 10.1016/j.ophtha.2005.04.020 16051363

[B23] Shields C. L. Furuta M. Thangappan A. Nagori S. Mashayekhi A. Lally D. R. (2009). Metastasis of UM millimeter-by-millimeter in 8033 consecutive eyes. *Arch. Ophthalmol.* 127 989–998. 10.1001/archophthalmol.2009.208 19667335

[B24] Shih H.-C. Liu E.-R. (2016). New quartile-based region merging algorithm for unsupervised image segmentation using color-alone feature. *Inform. Sci.* 342 24–36.

[B25] Sidhartha E. Gupta P. Liao J. Tham Y. C. Cheung C. Y. He M. (2014a). Assessment of iris surface features and their relationship with iris thickness in Asian eyes. *Ophthalmology* 121 1007–1012. 24405741 10.1016/j.ophtha.2013.11.028

[B26] Sidhartha E. Nongpiur M. E. Cheung C. Y. He M. Wong T. Y. Aung T. (2014b). Relationship between iris surface features and angle width in Asian eyes. *Invest. Ophthalmol. Vis. Sci.* 55 8144–8148. 25342619 10.1167/iovs.14-15402

[B27] Singh A. D. Bergman L. Seregard S. (2005). UM: epidemiologic aspects. *Ophthalmol. Clin. North Am.* 18 75–84, viii. 10.1016/j.ohc.2004.07.002 15763193

[B28] Singh A. D. Topham A. (2003). Incidence of UM in the United States: 1973–1997. *Ophthalmology* 110 956–961. 12750097 10.1016/S0161-6420(03)00078-2

[B29] Stang A. Ahrens W. Anastassiou G. Jöckel K. H. (2003). Phenotypical characteristics, lifestyle, social class and UM. *Ophthalmic Epidemiol.* 10 293–302. 14566630 10.1076/opep.10.5.293.17319

[B30] Stang A. Parkin D. M. Ferlay J. Jöckel K. H. (2005). International uveal melanoma incidence trends in view of a decreasing proportion of morphological verification. *Int. J. Cancer* 114 114–123. 15523698 10.1002/ijc.20690

[B31] Tomany S. C. Klein R. Klein B. E. Beaver Dam Eye Study (2003). The relationship between iris color, hair color, and skin sun sensitivity and the 10-year incidence of age-related maculopathy: the beaver dam eye study. *Ophthalmology* 110 1526–1533. 10.1016/s0161-6420(03)00539-6 12917167

[B32] Tomizuka T. Namikawa K. Higashi T. (2017). Characteristics of melanoma in Japan: a nationwide registry analysis 2011-2013. *Melanoma Res.* 27 492–497. 28609317 10.1097/CMR.0000000000000375

[B33] Vajdic C. M. Kricker A. Giblin M. McKenzie J. Aitken J. Giles G. G. (2002). Sun exposure predicts risk of ocular melanoma in Australia. *Int. J. Cancer* 101 175–182. 10.1002/ijc.10579 12209995

[B34] Wang L. Zhang K. Liu X. Long E. Jiang J. An Y. (2017). Comparative analysis of image classification methods for automatic diagnosis of ophthalmic images. *Sci. Rep.* 7:41545. 10.1038/srep41545 28139688 PMC5282520

[B35] Weis E. Shah C. P. Lajous M. Shields J. A. Shields C. L. (2006). The association between host susceptibility factors and UM: a meta-analysis. *Arch. Ophthalmol.* 124 54–60. 10.1001/archopht.124.1.54 16401785

[B36] Yang J. Zhang K. Fan H. Huang Z. Xiang Y. Yang J. (2019). Development and validation of deep learning algorithms for scoliosis screening using back images. *Commun. Biol.* 2:390. 10.1038/s42003-019-0635-8 31667364 PMC6814825

[B37] Zhang K. Li X. He L. Guo C. Yang Y. Dong Z. (2020). A human-in-the-loop deep learning paradigm for synergic visual evaluation in children. *Neural Netw.* 122 163–173. 10.1016/j.neunet.2019.10.003 31683144

[B38] Zhang K. Liu X. Jiang J. Li W. Wang S. Liu L. (2019). Prediction of postoperative complications of pediatric cataract patients using data mining. *J. Transl. Med.* 17:2. 10.1186/s12967-018-1758-2 30602368 PMC6317183

[B39] Zhang K. Liu X. Liu F. He L. Zhang L. Yang Y. (2018). An interpretable and expandable deep learning diagnostic system for multiple ocular diseases: qualitative study. *J. Med. Internet Res.* 20:e11144. 10.2196/11144 30429111 PMC6301833

[B40] Zhang Y. Li F. Yuan F. Zhang K. Huo L. Dong Z. (2020). Diagnosing chronic atrophic gastritis by gastroscopy using artificial intelligence. *Dig. Liver Dis.* 52 566–572. 10.1016/j.dld.2019.12.146 32061504

